# Leveraging AI to Evaluate Minimal Residual Disease Endpoint Surrogacy in Multiple Myeloma

**DOI:** 10.1158/2767-9764.CRC-25-0393

**Published:** 2026-05-25

**Authors:** Zexin Ren, Zixuan Zhao, Andrew J. Cowan, Will Ma, En Xie, Qian Shi

**Affiliations:** 1 George Washington University, Washington, District of Columbia.; 2 BC Cancer, University of British Columbia, Vancouver, Canada.; 3 HopeAI, Inc., Princeton, New Jersey.; 4 Mayo Clinic, Rochester, Minnesota.

## Abstract

Minimal residual disease (MRD) has been endorsed by the FDA Oncology Drugs Advisory Committee as an endpoint for accelerated approval in multiple myeloma based on individual patient data collected from randomized trials. However, emerging data from recent trials were not included. A novel artificial intelligence (AI)–assisted framework is proposed, which automates information identification and extraction, providing up-to-date analyses that confirm moderate trial-level and strong individual patient–level associations between MRD− rates at suspected complete response (MRD-CR) and survival endpoints in multiple myeloma. Specifically, this study utilized an AI-assisted framework that identifies relevant studies and filters critical information to analyze published data via 2 independent objectives. First, we examined the trial-level association using the coefficients of determination (R^2^) and its 95% confidence intervals (CI) based on published statistics of treatment effects on MRD and various endpoints. Next, we generated synthetic individual patient data with covariates through AI-curated tools to estimate the individual-level association. The AI tool searched for eligible randomized clinical trials (RCT). A total of 20 two-arm comparisons from 19 RCTs were analyzed. Trial-level analysis showed an *R*^2^ of 0.71 (95% CI, 0.52–0.89) pooling disease subpopulations. Furthermore, AI techniques were applied to create synthetic individual data, combining information extracted from Kaplan–Meier curves and subgroup analyses from published literatures. Using generated synthetic data, we estimated the individual-level correlation between MRD-CR rates and progression-free survival outcomes with a bivariate copula model and calculated a global odds ratio of 7.28 (95% CI, 5.60–8.95).

**Significance:**

(i) A novel AI-assisted framework is proposed, which automates information identification and extraction, providing rapid up-to-date analyses. (ii) Moderate trial-level and strong individual patient–level associations between MRD and various clinical endpoints in multiple myeloma are confirmed.

## Introduction

Significant advancements in the treatment of multiple myeloma over recent decades have led to substantially improved long-term outcomes. Recent reports indicate that progression-free survival (PFS) in newly diagnosed multiple myeloma now exceeds 4 years (1, 2). Additionally, some studies have observed nearly 100% objective response rates (ORR) in both treatment and control groups (2–4). The extended PFS duration necessitates large trials with prolonged follow-up periods, which hinders the development of new therapies and delays drug approval and patient access to effective multiple myeloma treatments. The very high ORR in control groups makes it impossible to demonstrate meaningful improved ORR in treatment groups and has become a less appealing endpoint for multiple myeloma trials. Minimal residual disease (MRD) has emerged as a promising endpoint associated with improved PFS and overall survival (OS) and has been recently endorsed by the FDA Oncology Drugs Advisory Committee for accelerated approval of new agents in multiple myeloma (5).

Despite the promise of MRD and other surrogate endpoints, the treatment options in multiple myeloma are evolving rapidly, with new immunomodulatory agents, proteasome inhibitors, and monoclonal antibodies frequently entering clinical practice. With rapid drug development, the previously validated surrogate endpoint may require reevaluation to reconfirm their surrogacy, especially if the new drugs involve changes in mechanisms of action. When additional trials become matured and/or published, previous surrogacy analyses may not fully capture the data from latest trials. Furthermore, reevaluation may be required because of the changes in standards of care or prolonged survival of the same standard-of-care treatment over time. On the other hand, regulatory bodies increasingly rely on surrogate endpoints to accelerate drug approval; thus, continuously demonstrating that MRD (and other potential surrogate endpoints) accurately reflects long-term clinical benefit remains essential. Repeatable updated analyses—conducted timely after earlier surrogacy-based findings—can clarify whether these endpoints still predict meaningful outcomes and guide both clinical decision-making and future drug development in multiple myeloma. However, a proper surrogacy analysis based on individual patient data from a large collection of completed randomized clinical trials (RCT) is time consuming. Innovative tools and methods to allow rapid surrogacy analysis or updated analysis are urgently needed.

This study introduces an artificial intelligence (AI)–assisted, expert-in-the-loop framework, designed to efficiently identify relevant studies and filter critical information for complex medical analysis objectives. As an illustration, we applied our framework to 2 equally important objectives: (i) estimating the trial-level associations between MRD− rates at suspected complete response (MRD-CR) endpoint and PFS/OS and (ii) generating novel synthetic individual patient data (IPD) via AI tools to assist the estimations of the individual patient–level association between MRD-CR endpoint and PFS/OS in the absence of the real IPD.

## Materials and Methods

### Study extraction and selection

RCTs, in multiple myeloma, published as full-length articles in PubMed and/or abstracts in major meetings such as American Society of Clinical Oncology, American Society of Hematology, European Hematology Association, and International Myeloma Society, spanning from January 1, 2010, to May 29, 2024, were systematically identified using AI. A systematic AI workflow (with illustrations) for extracting publications is provided in Supplementary Figs. S11 and S12. All 3 subpopulations, newly diagnosed transplant eligible (NDTE), newly diagnosed transplant ineligible (NDTI), and relapsed or refractory multiple myeloma (RRMM), were considered. For both objectives, studies were required to have more than 50 patients per treatment arm and the MRD negativity was classified at a minimum sensitivity threshold of $10^{-5}$, regardless of the testing methods of multiparametric flow cytometry (MFC), next-generation flow cytometry (NGF), or next-generation sequencing (NGS). In this application, the MRD-CR rate was defined as the proportion of patients achieving MRD negativity at a 10^−5^ threshold after suspected CR among patients who were randomized.

To be included in the analyses for the first objective, eligible RCTs were required to report the following statistics: (i) median PFS and/or median OS and/or survival rates at certain timepoints, (ii) MRD-CR rate by treatment arms, (iii) hazard ratios (HR) of and/or OS HR comparing PFS and/or OS between treatment groups. Additionally, the subsequent publications associated with each RCT, i.e., reporting data beyond the original primary trial endpoint(s), were screened for the eligibility to enter the analysis of the second objective. To be included in the analyses of the second objective, either Kaplan–Meier (KM) plots stratified by MRD-CR status were shown directly or KM plots of the intention-to-treat population with MRD-CR status included in the subgroup analysis were reported in the article(s).

A total number of 23 RCTs were identified. Among the identified RCTs, the DETERMINATION trial (NCT01208662; ref. 2) reported only the MRD− rate (not MRD-CR rate). The PLEIADES trial (NCT03412565; ref. 6) did not provide PFS/OS information. ICARIA-MM (NCT02990338; ref. 7) and KarMMA-3 (NCT03651128; ref. 8) reported MRD-CR rates in the control groups of 0% and 0.8%, respectively, which were deemed as outliers because the calculated ORR will be extremely large [log (ORR) = +inf for ICARIA-MM and log (ORR) = 3.4 for KarMMA-3]. Given the other data points [mean of log (ORR) ≈ 1.2], a log (ORR) of 3.4 is a high leverage point (9). Thus, they are excluded from the analysis of MRD− rate versus PFS/OS in the first objective. Therefore, the number of studies expanded from 15 trials in previous literature-based meta-analysis (10) to 19 RCTs with 20 two-arm comparisons, encompassing 8 NDTE, 5 NDTI, and 7 RRMM two-arm comparisons [one NDTE study STaMINA (11) has three arms and is therefore separated into 2 two-arm trials]. Study characteristics of 20 two-arm comparisons, including trial name and registration identification, disease subpopulation, MRD-CR rates, median progression free survival (mPFS), PFS/OS HRs with 95% confidence intervals (CI), and details of MRD assessment times and methods, were systematically extracted. Only the PFS data are presented in Table 1.

**Table 1.** Included trial information.

Trial	NCT ID	Disease	Method	Drug (sample size)	MRD-CR	mPFS	PFS HR	Assessment time
ATLAS (12)	NCT02659293	NDTE	NGS ($10^{-5}$)	KRd (*n* = 93) vs. R (*n* = 87)	53% vs. 31%	59.1 vs. 41.4	0.51	After cycle: 6 months
CASSIOPEIA (3)	NCT02541383	NDTE	NGS ($10^{-5}$)	D-VTd (*n* = 543) vs. VTd (*n* = 542)	34% vs. 20%	Inf vs. Inf	0.47	100 days after ASCT (4 cycle + 100 days)
GEM2012MENOS65 (13)	NCT01916252	NDTE	NGF ($10^{-6}$)	VRD-BuMel (*n* = 182) vs. VRD-Mel (*n* = 104)	58% vs. 55%	Inf vs. 75.3	0.88	At day 100 after HDT-ASCT and after consolidation ∼ 6 months
GRIFFIN (4)^a^	NCT02874742	NDTE	NGS ($10^{-5}$)	D-RVd (*n* = 104) vs. RVd (*n* = 103)	62% vs. 27%	Inf vs. Inf	0.45	At first evidence of suspected CR, VGPR, or suspected dara interference (no specific timepoint available)
PERSEUS (14)	NCT03710603	NDTE	NGS ($10^{-5}$)	D-VRd (*n* = 355) vs. VRd (*n* = 354)	75% vs. 48%	Inf vs. Inf	0.42	At suspected ≥CR (at 12, 18, 24, 30, and 36 months after cycle 1 day 1 and yearly thereafter)
TOURMALINE-MM3 (15)	NCT02181413	NDTE	MFC ($10^{-5}$)	Ixazomib (*n* = 395) vs. placebo (*n* = 261)	26% vs. 18%	26.5 vs. 21.3	0.83	At suspected ≥CR, VGPR at study entry, cycles 13 and 26 ∼(12 months and 24 months)
STaMINA (11)	NCT01109004	NDTE	NGS ($10^{-5}$)	Single Auto (144) vs. Auto + RVd (92)	75% vs. 78%	​	0.695	At 12 months
STaMINA (11)	NCT01109004	NDTE	NGS ($10^{-5}$)	Tandem Auto (55) vs. Auto + RVd (92)	85% vs. 78%	​	0.985	At 12 months
CLARION (16)^a^	NCT01818752	NDTI	NGF ($10^{-6}$)	KMP (*n* = 478) vs. VMP (*n* = 477)	5% vs. 5%	22.3 vs. 22.1	0.91	At first suspected ≥CR and/or EOT ∼(12 months)
TOURMALINE-MM2 (17)	NCT01850524	NDTI	NGS ($10^{-5}$)	Ixazomib (*n* = 351) vs. placebo (*n* = 354)	15% vs. 7%	35.3 vs. 21.8	0.83	At first suspected ≥CR and/or EOT ∼(12 months)
ALCYONE (18)^a^	NCT02195479	NDTI	NGF ($10^{-5}$)	D-VMP (*n* = 350) vs. VMP (*n* = 356)	28% vs. 7%	36.5 vs. 19.3	0.42	At first suspected ≥CR or at 18 months for patients ≥CR
MAIA (19)	NCT02252172	NDTI	NGF ($10^{-5}$)	DRd (*n* = 368) vs. Rd (*n* = 369)	31% vs. 10%	Inf vs. 31.9	0.53	At CR suspected (no specific timepoint available)
OCTANS (20)^a^	NCT03217812	NDTI	MFC ($10^{-5}$)	D-VMP (*n* = 146) vs. VMP (*n* = 74)	30% vs. 7%	Inf vs. 18.2	0.43	At the time of CR/sCR confirmation and 12, 18, 24, and 30 months after the first dose of study treatment for patients who maintained CR/sCR
POLLUX (21)^a^	NCT02076009	RRMM	NGS ($10^{-5}$)	DRd (*n* = 286) vs. Rd (*n* = 283)	33% vs. 7%	44.5 vs. 17.3	0.44	At CR/sCR confirmation and 12, 18, 24, and 30 months after first dose for patients who maintained CR/sCR
CASTOR (22)^a^	NCT02136134	RRMM	NGS ($10^{-5}$)	D-Vd (*n* = 251) vs. Vd (*n* = 247)	15% vs. 2%	16.7 vs. 7.1	0.31	At suspected ≥CR and 3 and 6 months after suspected ≥CR
BOSTON (23)	NCT03110562	RRMM	NGS ($10^{-5}$)	SVd (*n* = 195) vs. Vd (*n* = 207)	5% vs. 4%	13.9 vs. 9.5	0.70	At suspected ≥CR at cycles 9 and 15 ∼(9 months and 15 months)
CANDOR (24)	NCT03158688	RRMM	NGS ($10^{-5}$)	KdD (*n* = 312) vs. Kd (*n* = 154)	22% vs. 8%	28.4 vs. 15.2	0.64	At suspected ≥CR (at 12 months)
APOLLO (25)	NCT03180736	RRMM	NGS ($10^{-5}$)	DaraPD (*n* = 151) vs. PD (*n* = 153)	9% vs. 2%	12.4 vs. 6.9	0.63	At the time of suspected CR or stringent CR; at 6, 12, 18, and 24 months; and every 12 months after achieving CR or stringent CR
IKEMA (26)^a^	NCT03275285	RRMM	NGS ($10^{-5}$)	Isa-Kd (*n* = 179) vs. Kd (*n* = 123)	26% vs. 12%	35.7 vs. 19.2	0.58	At suspected ≥CR; at 6, 12, 18, and 24 months; and every 12 months after achieving CR/sCR until PD
CARTITUDE-4 (27)	NCT04181827	RRMM	NGS ($10^{-5}$)	Cilta-cel (*n* = 208) vs. PVd/DPd (*n* = 211)	61% vs. 16%	Inf vs. 11.8	0.26	At any time after VGPR (no specific timepoint available)

The assessment times for MRD-CR rates are assumed to be at suspected CR or better during the study.

Abbreviations: ASCT, Autologous stem cell transplantation; Auto, shorthand for ASCT; Cilta-cel, ciltacabtagene autoleucel; Dara, daratumumab; DaraPD, daratumumab + pomalidomide + dexamethasone; DPd, Daratumumab + Pomalidomide + dexamethasone; DRd, Daratumumab + Lenalidomide + dexamethasone; D-RVd, daratumumab + lenalidomide + bortezomib + dexamethasone; D-Vd, Daratumumab + Bortezomib + dexamethasone; D-VMP, Daratumumab + Bortezomib + Melphalan + Prednisone; D-VRd, daratumumab + bortezomib + lenalidomide + dexamethasone; D-VTd, daratumumab + bortezomib +thalidomide + dexamethasone; EOT, end of treatment; HDT, High-dose therapy; Inf, infinity not reached; Isa-Kd, isatuximab + carfilzomib + dexamethasone; Kd, carfilzomib + dexamethasone; KdD, carfilzomib + dexamethasone + daratumumab; KdD, Carfilzomib + dexamethasone + Daratumumab; KMP, carfilzomib + melphalan + prednisone; KRd, dexamethasone + lenalidomide + carfilzomib; NCT, National clinical trial identifier; PVd, Pomalidomide + Bortezomib + dexamethasone; sCR, stringent complete response; SVd, selinexor + bortezomib + dexamethasone; SVd, Selinexor + Bortezomib + dexamethasone; VGPR, very good partial response; VMP, bortezomib + melphalan + prednisone; VRD-BuMel, bortezomib + lenalidomide + dexamethasone + busulfan + melphalan; VRD-Mel, Bortezomib + Lenalidomide + Dexamethasone, combined with Melphalan.

a Follow-up studies available.

Additionally, among these, 7 (1 NDTE, 3 NDTI, 3 and RRMM) studies have required additional information for the analysis of the second objective. The asterisk (*) denotes studies with follow-up studies that would be used in the second objective.

### Statistical methods

For the first objective, AI tools were used to extract relevant summary statistics from identified eligible studies, followed by manual verification by 2 independent investigators (see Supplementary Fig. S11) and discussions beyond for details. Trial-level associations were quantified using coefficients of determination (*R*^2^) with 95% CIs, obtained from weighted least squares methods weighted by trial sample sizes. Alternative weight, such as inverse variances, was also considered. Subgroup analyses were carried out within newly diagnosed multiple myeloma, NDTI, and RRMM subpopulations, separately. The criteria for interpreting *R*^2^ were predefined as poor (*R*^2^ < 0.4), moderate (0 ≤ *R*^2^ < 0.8), and strong correlation (*R*^2^ ≥ 0.8).

For the second objective, we aimed to conduct an individual-level surrogacy analysis using the bivariate copula model, which requires IPD from all published studies. Obtaining the true IPD for each study is challenging because of privacy and proprietary concerns. To overcome this barrier, we utilized a novel AI-powered synthetic data–generating algorithm (SynthIPD) to generate IPD that mimics the original data (arXiv 2509.16466). In brief, SynthIPD captures scalable vector graphics from the published clinical trial article, digitizes the coordinates of KM survival plots, and reproduces the survival endpoints, failure status, and treatment indication for each patient. Additionally, it can generate synthetic covariate (e.g., MRD-CR status) information that best matches the true covariate distribution. Compared with the off-the-shelf digitization algorithm IPDfromKM (28), which requires manual extraction, the new method is more flexible (in the sense that it can incorporate covariates) and more accurate (in terms of capturing the exact failure and censoring times). An example of a generated data set is presented in Table 2 using the ALCYONE study (18).

**Table 2.** Example header of the synthetic data generated for ALCYONE study.

ID	Study	PFS	Status	Treatment	MRD	Population
1	ALCYONE	8.352849	0	D-VMP	Neg	NDTI
2	ALCYONE	9.445576	1	D-VMP	Neg	NDTI
3	ALCYONE	9.706880	1	D-VMP	Neg	NDTI
4	ALCYONE	0.090576	0	D-VMP	Pos	NDTI
5	ALCYONE	16.51267	0	VMP	Neg	NDTI

The third column is the synthetic PFS; the fourth column is the censoring status, where we denote by 1 to be an event and 0 to be a censoring; the fifth column represents treatment received; the sixth column represents MRD-CR status, and the last column shows the disease population.

Abbreviations: D-VMP, Daratumumab + Bortezomib + Melphalan + Prednisone; Neg, negative; Pos, positive; VMP, bortezomib + melphalan + prednisone.

The individual patient–level association between MRD-CR status and PFS outcomes in patients with multiple myeloma was calculated using only the generated synthetic IPD. Bivariate Plackett copula models were used to estimate the individual-level association, estimated by global odds ratio (OR; ref. 29), using AI-generated synthetic IPD. The individual patient–level surrogacy was also evaluated within each disease population separately. The global OR can be interpreted as the ORs for a patient being alive and progression free beyond a timepoint when comparing patients with MRD-CR versus those without MRD negativity, adjusting for treatments. For example, a global OR of 2 indicates that the odds of being alive and progression free beyond a timepoint (given that the patient is known to be alive and progression free up to that point) for a patient achieving CR and MRD negativity is 2 times of the odds for a patient with positive MRD status. For global OR, a value higher than 1 indicates that patients who achieve MRD negativity (beyond CR) have a longer PFS outcome in general. The individual-level association is considered strong if the global OR is high (e.g., ≥3) and the 95% CI excludes 1.

## Results

### Objective 1: Trial-level association

For the first objective, trial-level coefficients of determination were calculated for (i) log (HR) PFS versus log (OR) MRD; (ii) log (HR) OS versus log (OR) MRD (see Supplementary Figs. S3 and S4); (iii) log (ORR) versus log (OR) MRD (see Supplementary Fig. S5); and (iv) log (HR) PFS/OS versus log (OR) MRD for each subgroup stratified by disease type (NDTE, NDTI, and RRMM; see Supplementary Fig. S6).

**Figure 1.**
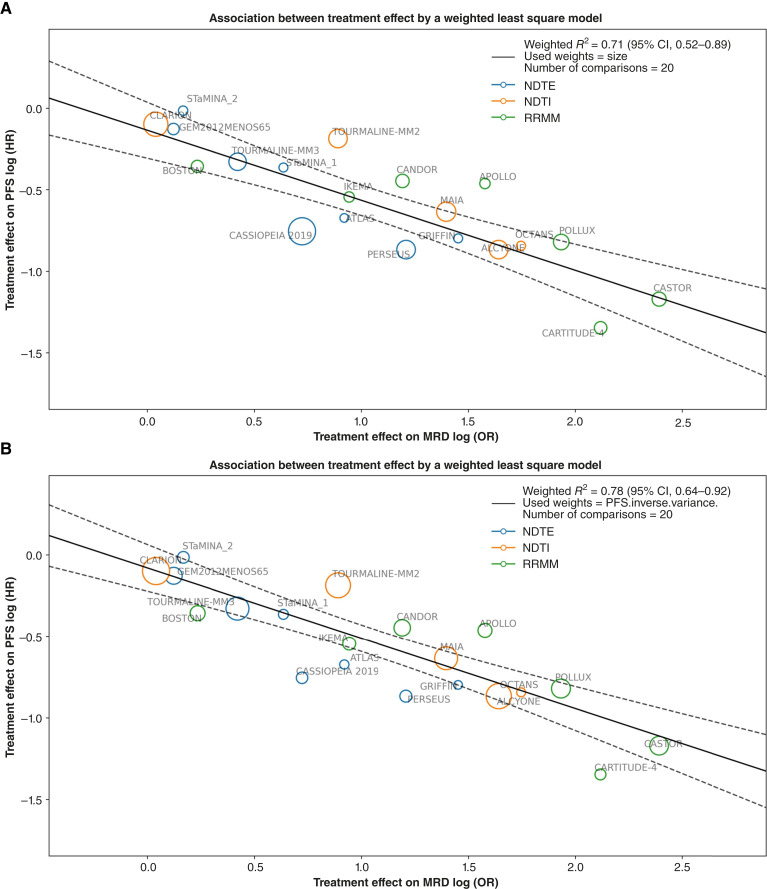
Weighted $R_{trial}^{2}$ in the aggregated analysis of 19 clinical trials. PFS HR and MRD-CR OR are natural log-transformed. The black solid lines are the fitted regression lines, and the black dotted lines are 95% CI bands. **A,** PFS log (HR) vs. MRD log (OR) weighted by sample size of each trial. **B,** PFS log (HR) vs. MRD log (OR) weighted by inverse variance of PFS HR.

Results for (i) are reported in Fig. 1A and Table 3 using sample sizes as weights. Additionally, results using inverse variance of PFS HR were reported in Fig. 1B and Table 3. The weighted trial-level coefficient of determination for (i) log (HR) PFS versus log (OR) MRD-CR observed in the aggregated analysis of the 20 two-arm comparisons was $R_{trial}^{2}$ = 0.71 (95% CI, 0.52–0.89). Sensitivity analyses using a leave-one-out approach showed $R_{trial}^{2}$ ranging from 0.63 to 0.80 (Supplementary Fig. S7). Another sensitivity analysis using 18 studies with sensitivity $10^{-5}$ only was conducted (i.e., excluding trials based on the 10^−6^ threshold; see Supplementary Figs. S1 and S2), showing moderate correlation $R_{trial}^{2}$ = 0.59 and 0.70, respectively, for the two different weights. Additional sensitivity analyses based on trials with different MRD assessment methods and assessment timings are reported (see Supplementary Figs. S8 and S9). Specifically, we performed sensitivity analyses across 3 MRD assessment methods—NGS, NGF, and MFC. In addition, a single sensitivity analysis focusing on MRD assessment timing was conducted, restricted to trials that assessed MRD at the time when patients were suspected to have achieved CR. Summarizing the results, the ORs of MRD− rates in multiple myeloma are moderately correlated with the HR for PFS. The result aligns with recent discoveries in (10) using 15 studies with reported $R_{trial}^{2}$ = 0.70 (0.41–0.98) and (30) using 13 studies with reported $R_{trial}^{2}$ = 0.53 (0.21–0.77).

**Table 3.** Trial-level $R^{2}$ and 95% CIs using weighted least squares with different disease populations and different weights.

Outcome	Disease population	Number of two-arm comparisons	$R^{2}$ (weights = sample sizes)	$R^{2}$ (weights = inverse variance)
Log (PFS HR)	NDTE	8	0.78 (0.61–0.95)	0.88 (0.78–0.98)
	NDTI	5	0.85 (0.74–0.97)	0.84 (0.71–0.96)
	RRMM	7	0.72 (0.50–0.93)	0.72 (0.51–0.93)
	Pooling 3 subpopulations	20	0.71 (0.52–0.89)	0.78 (0.64–0.92)
Log (OS HR)	NDTE	5	0.13 (<0.01–0.41)	0.31 (<0.01–0.65)
	NDTI	4	0.86 (0.76–0.96)	0.85 (0.75–0.96)
	RRMM	7	0.62 (0.36–0.89)	0.64 (0.39–0.9)
	Pooling 3 subpopulations	16	0.59 (0.35–0.84)	0.61 (0.37–0.85)

Results for (ii) are reported in Supplementary Fig. S3 with sample sizes as weights. The weighted trial-level $R_{trial}^{2}$ was 0.56 (0.31–0.82), showing a moderate correlation between OR for MRD-CR rates and HR for OS. Results using inverse variance of OS log (HR) are provided in Supplementary Fig. S4.

The subgroup analyses were conducted for NDTE, NDTI, and RRMM populations. For NDTE, $R_{trial}^{2}$ = 0.78 (0.61–0.95); for NDTI, $R_{trial}^{2}$ = 0.85 (0.74–0.97); and for RRMM, $R_{trial}^{2}$ = 0.72 (0.50–0.93). Notably, the NDTI subgroup exhibited a strong correlation with $R_{trial}^{2}$ > 0.8 and a lower CI bound greater than 0.6. The correlation, though very strong, included only 5 NDTI studies and should be interpreted with caution.

Trial-level surrogacy for OS was consistently weaker than that for PFS, particularly in NDTE populations (see, e.g., Table 3). This is expected as OS is a later endpoint that is influenced by postprogression therapies, treatment cross-over, and subsequent lines of therapy, all of which are not captured by MRD status and can attenuate trial-level associations. These effects are amplified in NDTE settings, where longer survival and the availability of effective therapies introduce additional heterogeneity in OS outcomes.

### Objective 2: Individual-level surrogacy analysis

The second task aimed to investigate the role of MRD-CR rates in long-term survival outcomes based on synthetic IPD. AI searched for updated publications on MRD-CR rates via KM curves stratified by MRD-CR status in the previous 18 studies. Among those studies, updated versions of 7 studies are available (GRIFFIN, ALCYONE, CLARION, OCTANS, POLLUX, CASTOR, and IKEMA): 1 NDTE, 3 NDTI, and 3 RRMM studies. Synthetic IPD (like in Table 2) were generated and pooled via the SynthIPD method for all 7 studies, resulting in 207, 1,149, and 1,372 observations for NDTE, NDTI, and RRMM populations, respectively. An illustration of the SynthIPD result for POLLUX study is given in Supplementary Fig. S10.

The global ORs, along with 95% CIs, are reported in Table 4. Pooling all 7 studies, the global OR was 7.28 (95% CI, 5.60–8.95). Pooling NDTI and RRMM populations, the global OR was 7.58 (95% CI, 5.80–9.37). When considering disease subpopulations separately, the global OR was 6.37 (95% CI, 4.32–8.41) and 9.21 (95% CI, 6.01–12.42), respectively. All results showed a very strong signal for the association between MRD-CR status and prolonged PFS outcomes.

**Table 4.** Reported estimates and CIs for $R^{2}$, copula $R^{2}$, and global OR for the 7 synthetic studies using weighted least squares.

Subset	Number of comparisons (number of patients)	Global OR (95% CI)
All studies	7 (2,728)	7.28 (5.60–8.95)
NDTI/RRMM	6 (2,521)	7.58 (5.80–9.37)
NDTI	3 (1,149)	6.37 (4.32–8.41)
RRMM	3 (1,372)	9.21 (6.01–12.42)

The individual-level and trial-level analyses address distinct questions. The global OR from the individual-level model quantifies the prognostic association between MRD negativity and PFS within patients, conditional on treatment. In contrast, the trial-level $R^{2}$ evaluates whether across trials larger treatment effects on MRD translate into larger treatment effects on PFS. In fact, strong individual-level association does not imply strong trial-level surrogacy, as the latter is sensitive to between-trial heterogeneity in treatment mechanisms. These 2 different approaches provide complementary evidence, informing the suitability of MRD as a surrogate endpoint for regulatory and decision-making purposes.

## Discussion

Our trial-level analysis, which yielded $R_{trial}^{2}$ = 0.71 (0.52–0.89) and $R_{trial}^{2}$ = 0.78 (0.64–0.92) for the association between log (HR) for PFS and the log OR for MRD-CR rate, confirms that although there is a moderate correlation, the threshold ($R_{trial}^{2}$ > 0.8) for validating MRD-CR as a surrogate for mPFS is not met. These findings are consistent with prior research (10) yet also reflect refinements made by excluding 2 studies that did not meet our eligibility criteria and incorporating 5 additional studies.

Moreover, our individual-level analysis, using the newly proposed SynthIPD technique, demonstrated that MRD-CR is associated with prolonged survival outcomes, with results closely mirroring those obtained from real data (with summary statistic errors typically within 2%). Using the bivariate copula model (29) for surrogacy evaluation, it was shown that in both subgroups MRD− status was associated with prolonged survival outcomes compared with MRD+ groups.

Our study introduces a novel, AI-driven framework that represents a significant advancement over conventional meta-analyses and pooled IPD analyses. Traditional methodologies often require extensive time investments, ranging from 6 months for meta-analyses (31) to years for pooled IPD analyses. On the other hand, by leveraging AI with an expert-in-the-loop design, as well as AI-generated SynthIPD, our approach was able to complete robust and comprehensive statistical analyses in less than 2 weeks. This method not only dramatically reduces time consumption but also ensures that the analysis is both comprehensive and statistically robust by integrating the most current clinical evidence available.

Despite these advancements, several limitations warrant consideration. First, our analysis is entirely literature based, and differences in treatment regimens, MRD-CR assessment timepoints, detailed derivation of MRD-CR status, and methodologies across studies introduce heterogeneity, which is possibly the most common drawback in aggregated analyses of MRD in multiple myeloma. Second, privacy and proprietary concerns in medical research precluded a direct individual-level assessment of the relationship between MRD-CR rate and PFS/OS. Although our secondary objective, based on synthetic data, aligns with contemporary surrogacy evaluation methods, further validation with real, pooled IPD is necessary. Lastly, our analysis is constrained by the limited number of trials (19 in total, with only 7 contributing to the second objective and only 1 NDTE trial), which may affect the generalizability and reliability of our findings.

In summary, our study underscores the clinical relevance of MRD-CR in multiple myeloma and demonstrates that an AI-driven approach can substantially reduce the time required for rigorous surrogacy evaluation.

## Supplementary Material

Figure S1 Figure S1. The weighted R² trial in the aggregated analysis of 18 clinical trials with sensitivity level 10^−^5 only.

Figure S2 Figure S2. The weighted R² trial in the aggregated analysis of 18 clinical trials with sensitivity level as 10^−^5 only.

Figure S3 Figure S3. The weighted R² trial in the aggregated analysis of 16 clinical trials reporting OS information.

Figure S4 Figure S4. The weighted R² trial in the aggregated analysis of 16 clinical trials reporting OS information.

Figure S5 Figure S5. The weighted R² trial in the aggregated analysis of 16 clinical trials reporting ORR information.

Figure S6 Figure S6. The weighted R² trial in the subgroup analysis of 18 clinical trials reporting PFS information.

Figure S7 Figure S7. Leave-one-out association plot.

Figure S8 Figure S8. The weighted R² trial in the aggregated analysis of clinical trials stratified by MRD assessment method (NGS, NGF, and MFC).

Figure S9 Figure S9. The weighted R² trial in the aggregated analysis of 8 clinical trials with MRD assessed when patients achieve suspected CR.

Figure S10 Figure S10. Comparison between reported KM curves (truth) and digitized KM curves (via SVG by our algorithm) for the POLLUX study.

Figure S11 Figure S11. The AI workflow for screening trials.

Figure S12 Figure S12. A complete illustration of the agentic workflow for one iteration.

Descriptive notes for supplement Descriptive notes for supplement

## Data Availability

Data for trial-level association are provided in Table 1. Synthetic IPD data for individual association analysis are available from Z. Ren upon request.
