# Supplementary material for: Leveraging AI to Evaluate Minimal Residual Disease Endpoint Surrogacy in Multiple Myeloma
Source: Cancer Res Commun. 2026 May 25;6(5):1206–12. doi: 10.1158/2767-9764.CRC-25-0393 (PMC13200265; doi:10.1158/2767-9764.CRC-25-0393)
Supplement: Figure S1 — The weighted R² trial in the aggregated analysis of 18 clinical trials with sensitivity level 10−5 only. [file crc-25-0393_figure_s1_suppsf1.docx]

# Supplementary Figure S1


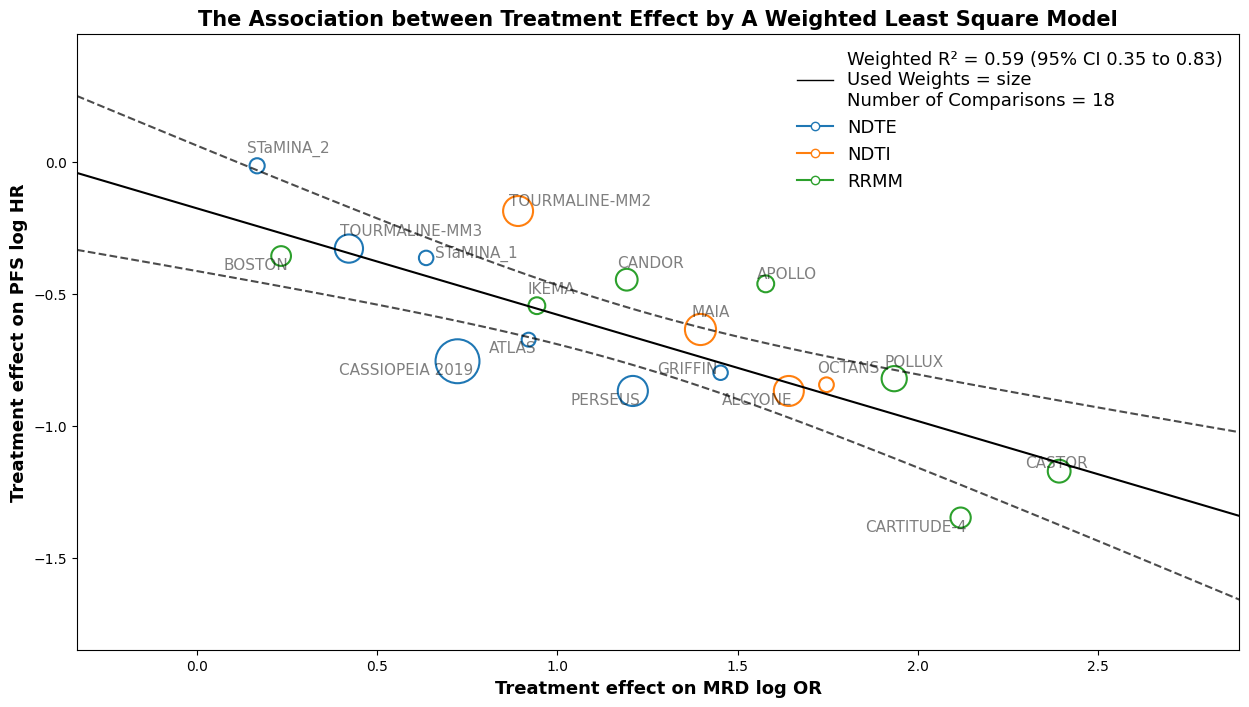


**Figure S1.** The weighted R²ₜᵣᵢₐₗ in the aggregated analysis of 18 clinical trials with sensitivity level 10⁻⁵ only. PFS HR and MRD-CR odds ratio are natural log transformed. The weights equal sample sizes. The black solid lines are the fitted regression lines and the black dotted lines are 95% confidence bands.
