# Supplementary material for: Leveraging AI to Evaluate Minimal Residual Disease Endpoint Surrogacy in Multiple Myeloma
Source: Cancer Res Commun. 2026 May 25;6(5):1206–12. doi: 10.1158/2767-9764.CRC-25-0393 (PMC13200265; doi:10.1158/2767-9764.CRC-25-0393)
Supplement: Figure S5 — The weighted R² trial in the aggregated analysis of 16 clinical trials reporting ORR information. [file crc-25-0393_figure_s5_suppsf5.docx]

# Supplementary Figure S5


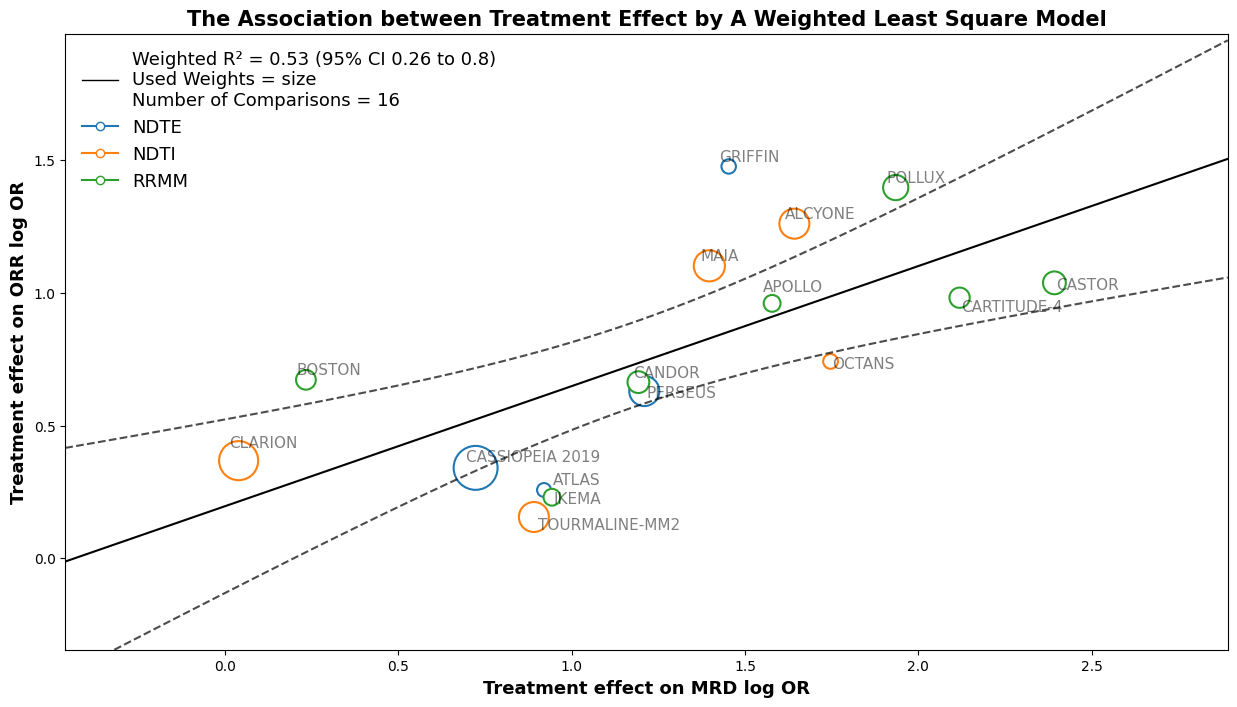


**Figure S5.** The weighted R²ₜᵣᵢₐₗ in the aggregated analysis of 16 clinical trials reporting ORR information. ORR OR and MRD-CR OR are natural log transformed. The weights equal sample sizes. The black solid lines are the fitted regression lines and the black dotted lines are 95% confidence bands.
