# Supplementary material for: Leveraging AI to Evaluate Minimal Residual Disease Endpoint Surrogacy in Multiple Myeloma
Source: Cancer Res Commun. 2026 May 25;6(5):1206–12. doi: 10.1158/2767-9764.CRC-25-0393 (PMC13200265; doi:10.1158/2767-9764.CRC-25-0393)
Supplement: Figure S7 — Leave-one-out association plot. [file crc-25-0393_figure_s7_suppsf7.docx]

# Supplementary Figure S7


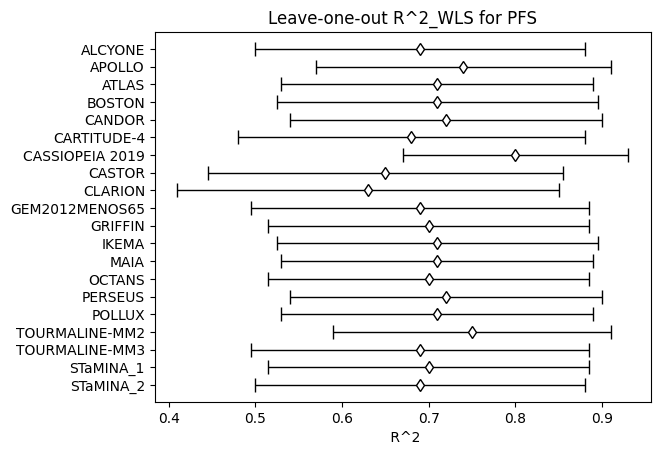


**Figure S7.** Leave-one-out association plot. The y-axis lists the names of excluded studies.
