# Supplementary material for: Leveraging AI to Evaluate Minimal Residual Disease Endpoint Surrogacy in Multiple Myeloma
Source: Cancer Res Commun. 2026 May 25;6(5):1206–12. doi: 10.1158/2767-9764.CRC-25-0393 (PMC13200265; doi:10.1158/2767-9764.CRC-25-0393)
Supplement: Figure S8 — The weighted R² trial in the aggregated analysis of clinical trials stratified by MRD assessment method (NGS, NGF, and MFC). [file crc-25-0393_figure_s8_suppsf8.docx]

# Supplementary Figure S8

**(a) PFS log(HR) versus MRD log(OR) weighted by sample size in trials assessed by NGS only.**


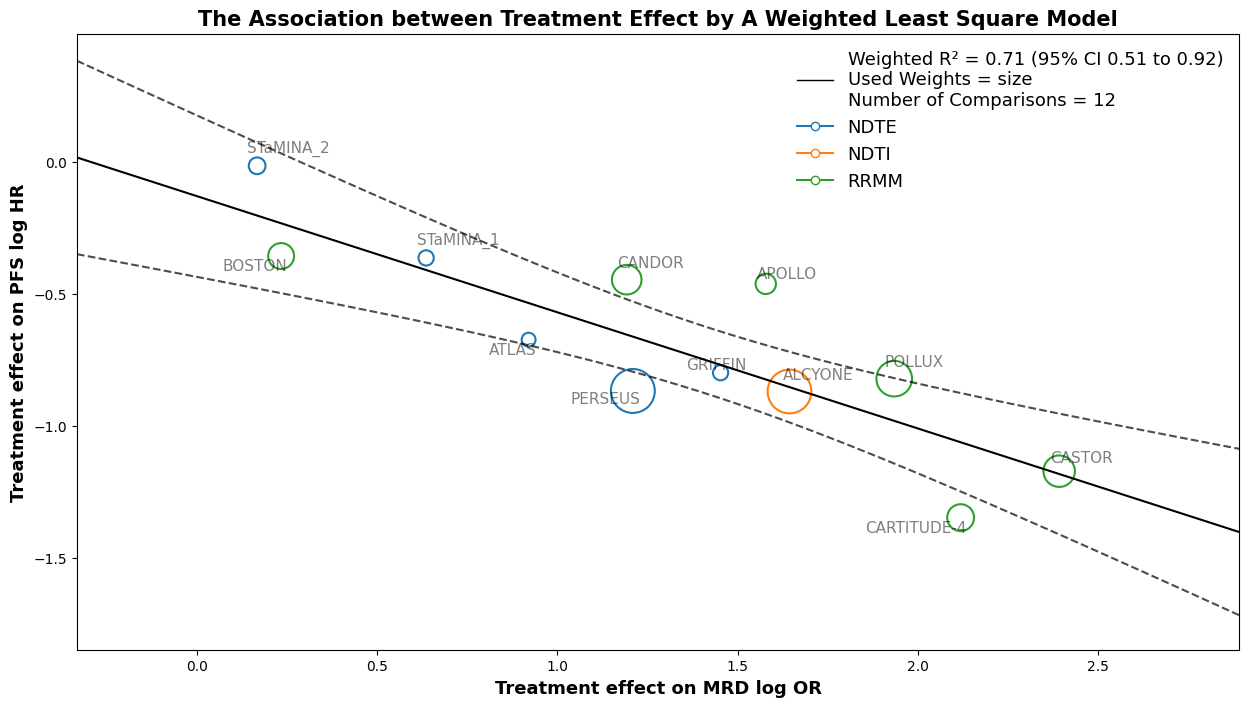


**(b) OS log(HR) versus MRD log(OR) weighted by sample size in trials assessed by NGS only.**


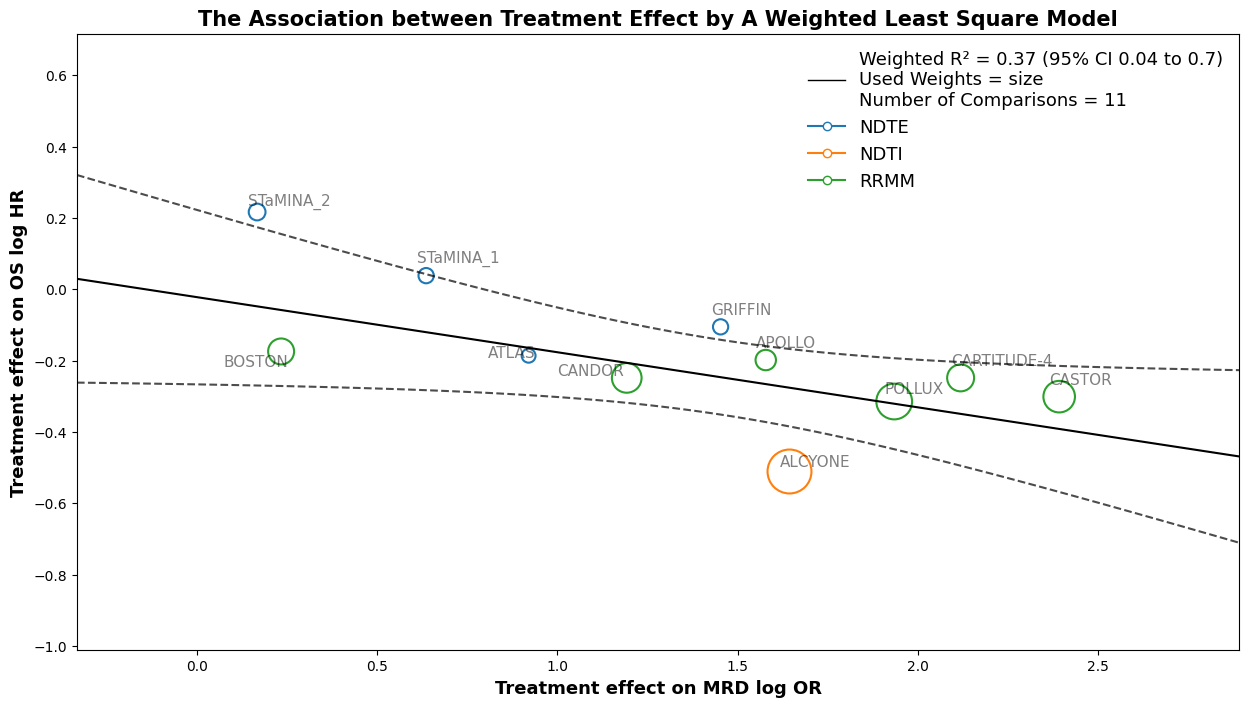


**(c) PFS log(HR) versus MRD log(OR) weighted by sample size in trials assessed by NGF only.**


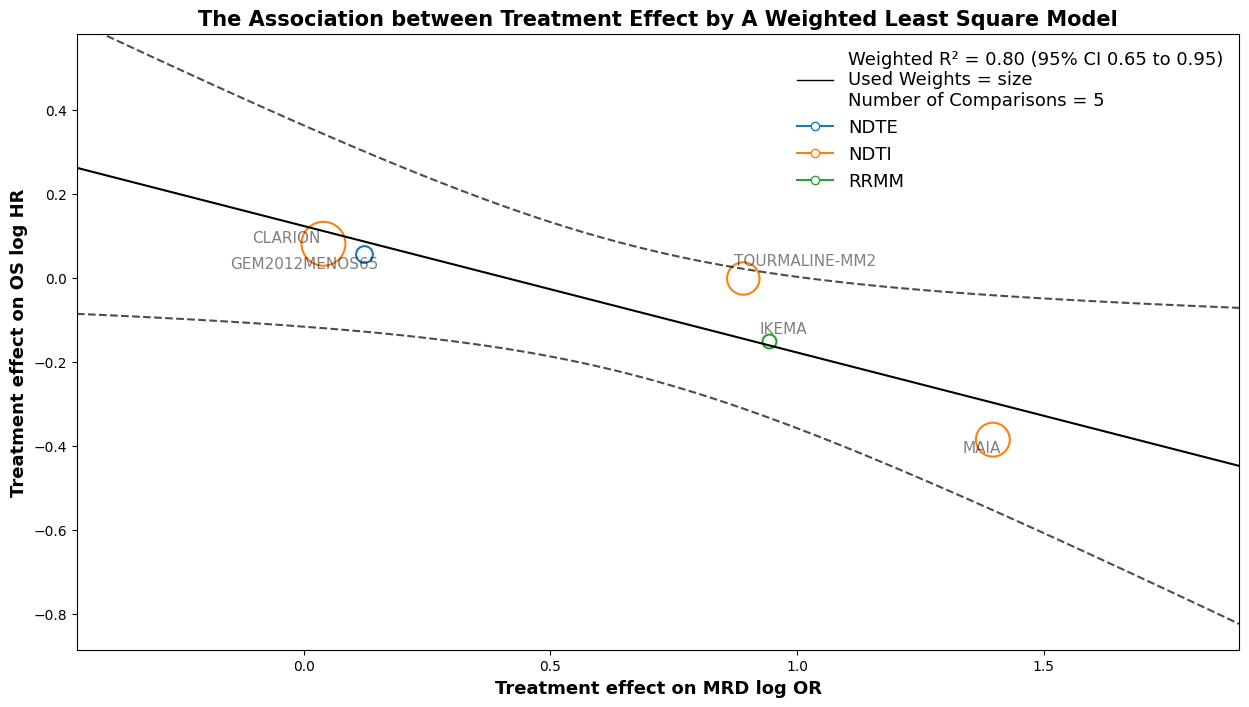


**(d) OS log(HR) versus MRD log(OR) weighted by sample size in trials assessed by NGF only.**


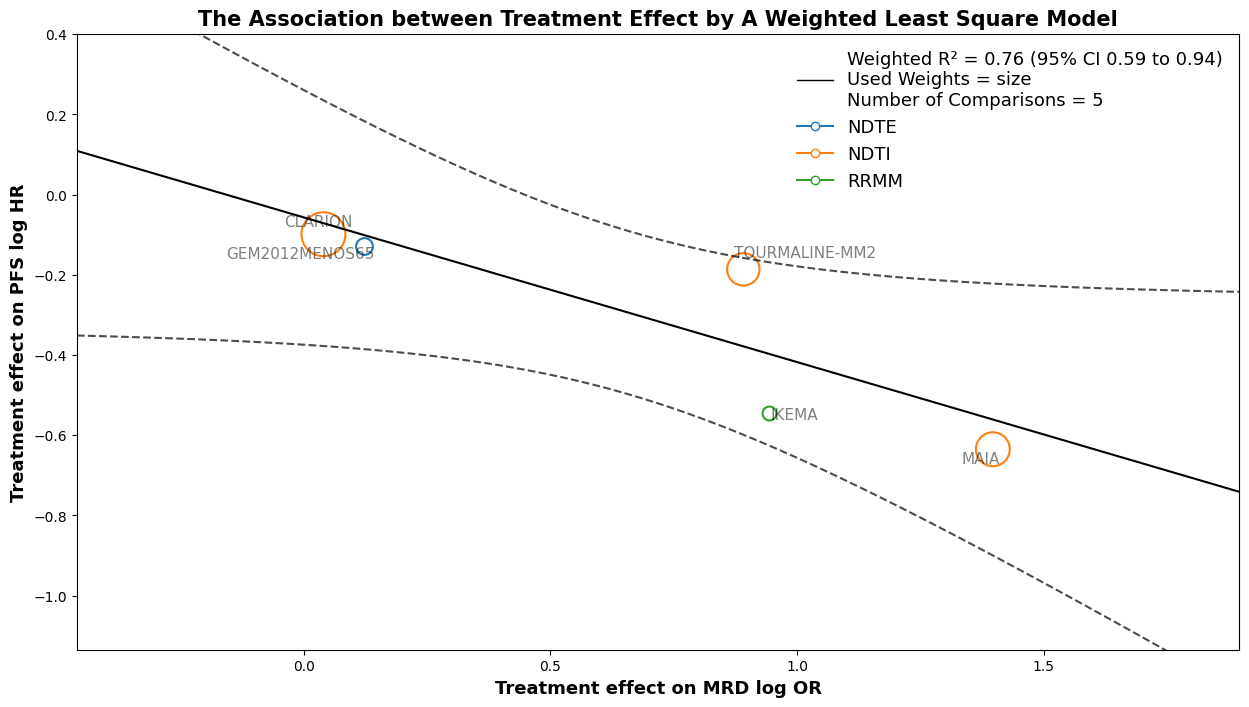


**(e) OS log(HR) versus MRD log(OR) weighted by sample size in trials assessed by MFC only.**


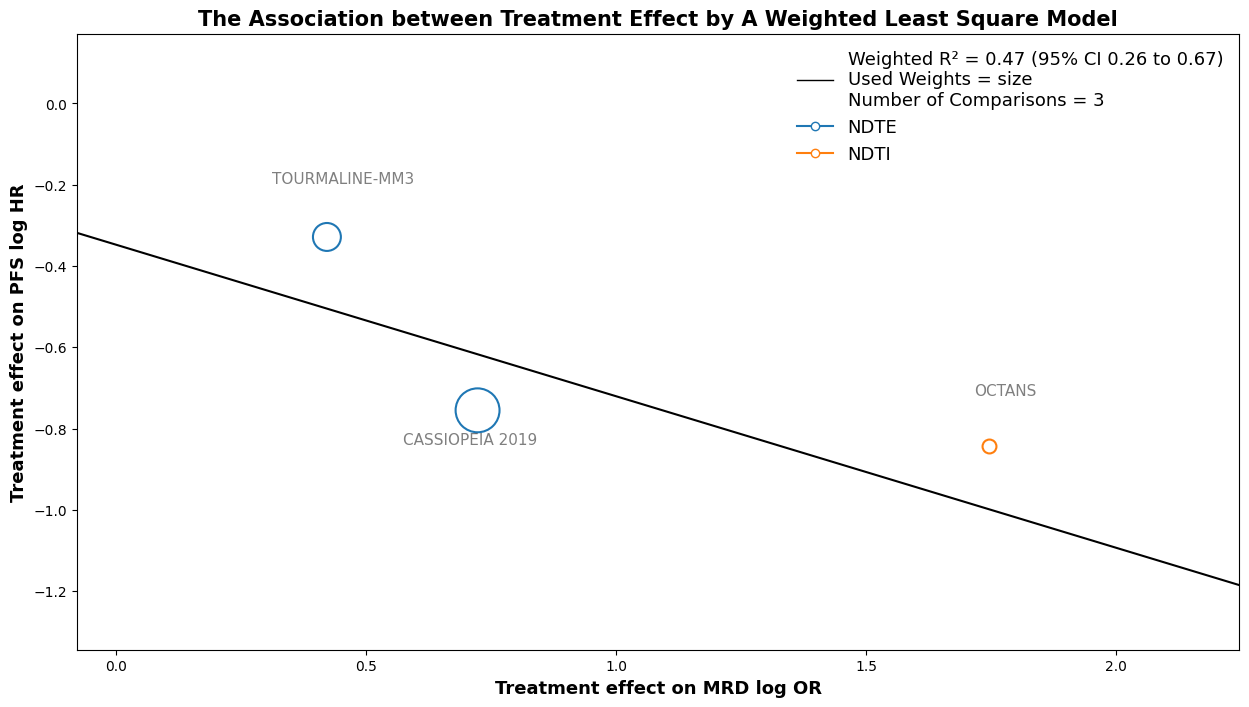


**Figure S8.** The weighted R²ₜᵣᵢₐₗ in the aggregated analysis of clinical trials stratified by MRD assessment method (NGS, NGF, and MFC). PFS HR, OS HR and MRD-CR odds ratio are natural log transformed. The weights equal sample sizes. The black solid lines are the fitted regression lines; the 95% confidence bands are too wide to fit the figure due to the low sample size.
