# Supplementary material for: Leveraging AI to Evaluate Minimal Residual Disease Endpoint Surrogacy in Multiple Myeloma
Source: Cancer Res Commun. 2026 May 25;6(5):1206–12. doi: 10.1158/2767-9764.CRC-25-0393 (PMC13200265; doi:10.1158/2767-9764.CRC-25-0393)
Supplement: Figure S9 — The weighted R² trial in the aggregated analysis of 8 clinical trials with MRD assessed when patients achieve suspected CR. [file crc-25-0393_figure_s9_suppsf9.docx]

# Supplementary Figure S9

**(a) PFS log(HR) versus MRD log(OR) weighted by sample size in trials assessed at suspected CR only.**


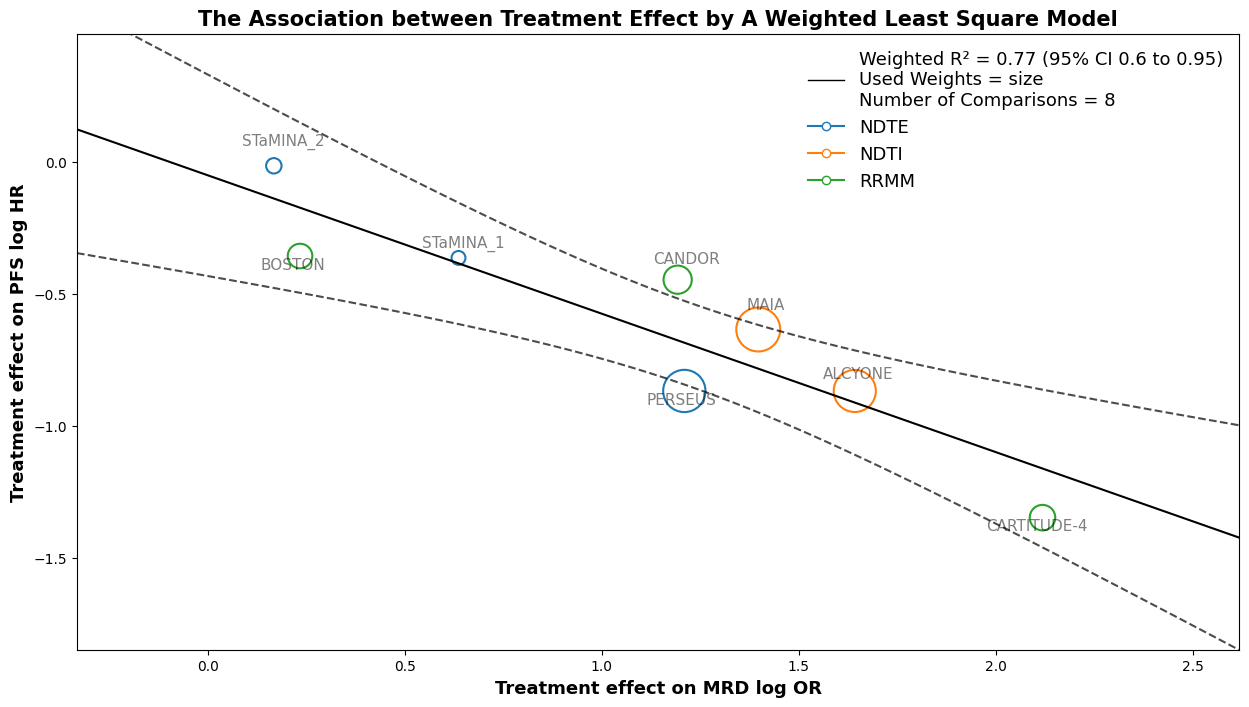


**(b) OS log(HR) versus MRD log(OR) weighted by sample size in trials assessed at suspected CR only.**


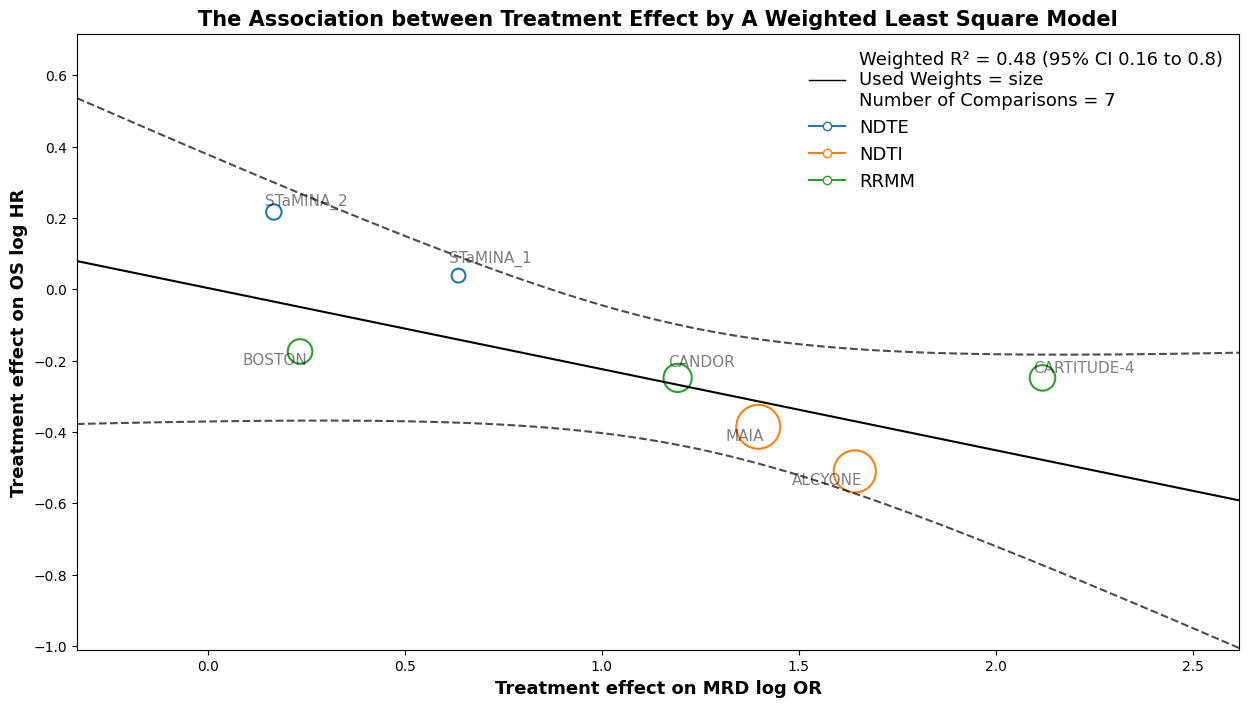


**Figure S9.** The weighted R²ₜᵣᵢₐₗ in the aggregated analysis of 8 clinical trials with MRD assessed when patients achieve suspected CR. PFS HR, OS HR and MRD-CR odds ratio are natural log transformed. The weights equal sample sizes. The black solid lines are the fitted regression lines and the black dotted lines are 95% confidence bands. We also conducted a sensitivity analysis based on the method and timing of MRD assessment. We observed that many studies evaluated MRD negativity at multiple time points, resulting in heterogeneous assessment schedules across trials. To ensure consistency, we restricted this analysis to the eight studies that explicitly reported MRD assessment at the time when patients were suspected to have achieved complete response. Of these eight trials, one study (PERSEUS) reported PFS results only; therefore, seven trials were included in the OS analysis. The results from this subgroup were consistent with those of the primary analysis. For example, the PFS association analysis yielded an R² = 0.77, comparable to the primary analysis result (R² = 0.71).
