# Supplementary material for: Leveraging AI to Evaluate Minimal Residual Disease Endpoint Surrogacy in Multiple Myeloma
Source: Cancer Res Commun. 2026 May 25;6(5):1206–12. doi: 10.1158/2767-9764.CRC-25-0393 (PMC13200265; doi:10.1158/2767-9764.CRC-25-0393)
Supplement: Figure S10 — Comparison between reported KM curves (truth) and digitized KM curves (via SVG by our algorithm) for the POLLUX study. [file crc-25-0393_figure_s10_suppsf10.docx]

# Supplementary Figure S10

**(a)**


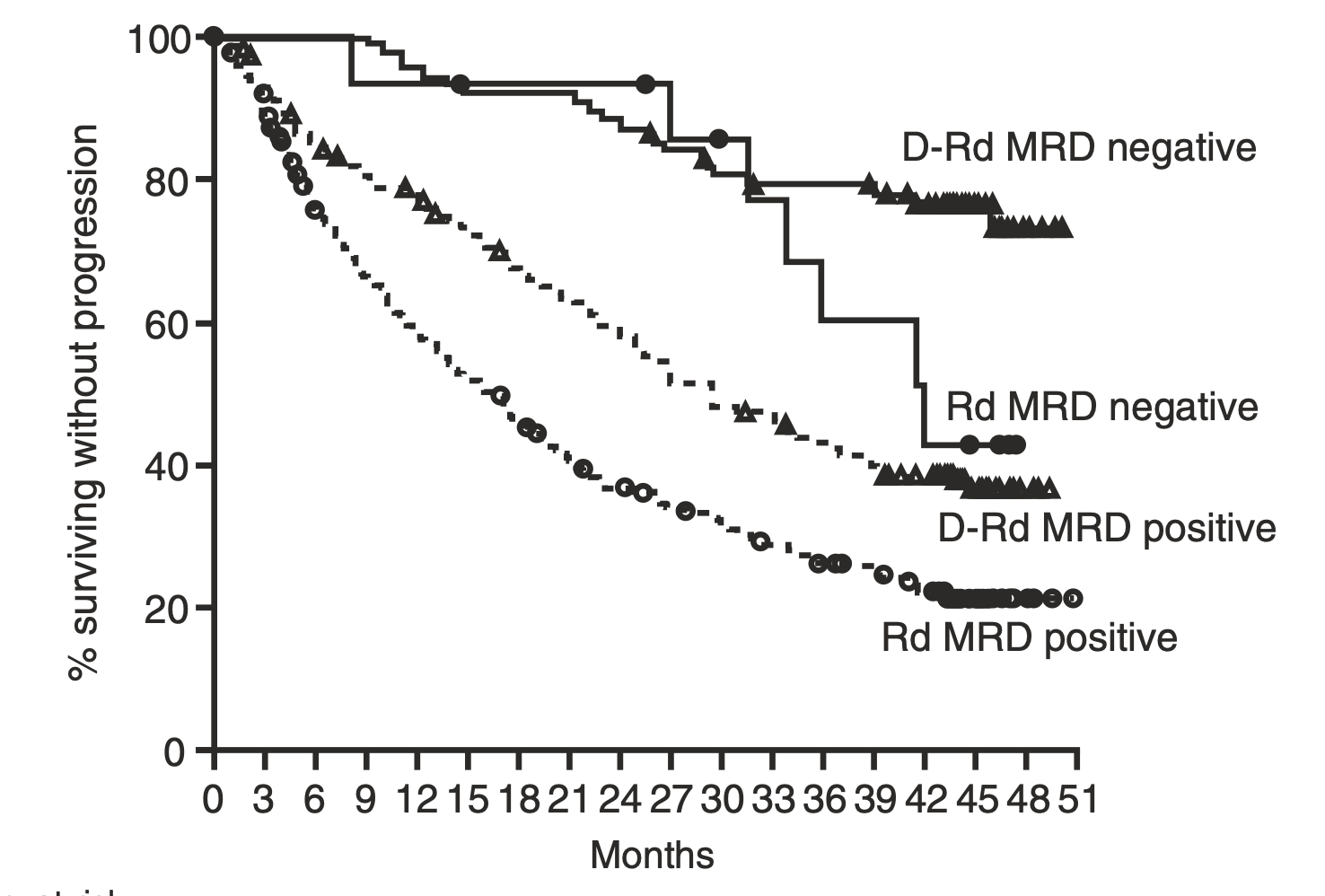


**(b)**


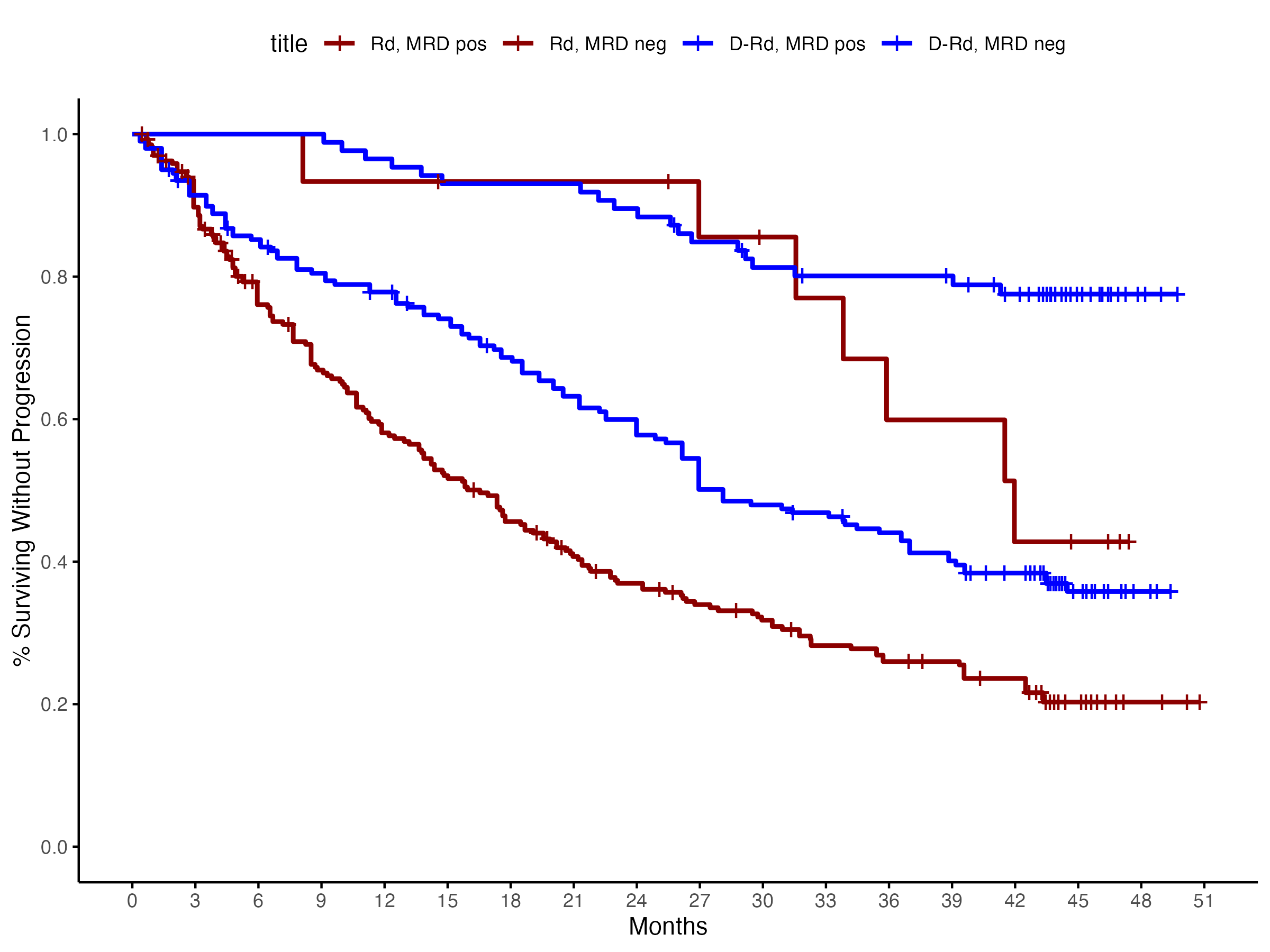


**Figure S10.** Comparison between reported KM curves (truth) and digitized KM curves (via SVG by our algorithm) for the POLLUX study. (a) The original plot provided by the POLLUX study; (b) The KM plot generated by SynthIPD. The drops indicating events are accurately captured along with the censorings.
