# Supplementary material for: Leveraging AI to Evaluate Minimal Residual Disease Endpoint Surrogacy in Multiple Myeloma
Source: Cancer Res Commun. 2026 May 25;6(5):1206–12. doi: 10.1158/2767-9764.CRC-25-0393 (PMC13200265; doi:10.1158/2767-9764.CRC-25-0393)
Supplement: Figure S11 — The AI workflow for screening trials. [file crc-25-0393_figure_s11_suppsf11.docx]

# Supplementary Figure S11


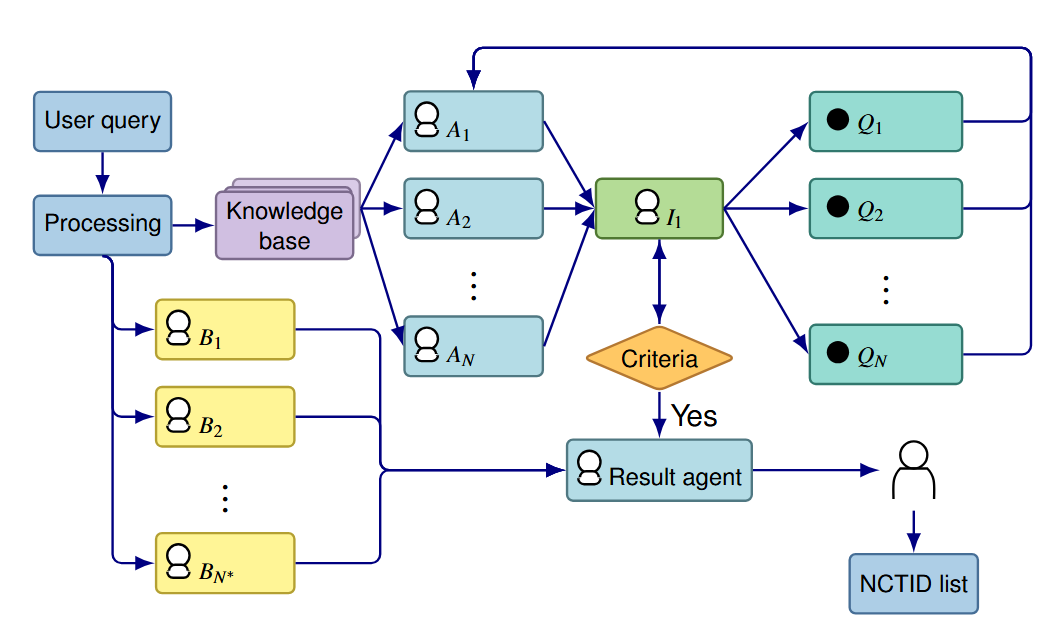


**Figure S11.** The AI workflow for screening trials. The AI agentic workflow shown in Supplementary Figure S11 is used to extract eligible trials satisfying the inclusion/exclusion criteria in the main paper. In this workflow, the query is processed by a query processing agent and passed to three independent reasoning agents (A1-A3) to evaluate whether the provided trials in the knowledge base (e.g., all MM trials on clinicaltrials.gov) satisfy the inclusion/exclusion criteria. The results by A1-A3 are passed to an inspector agent A4, which checks whether the previous agents reached consensus on deciding if the trial is eligible. If consensus is reached, the inspector passes the trial to the result agent; otherwise, the inspector provides targeted follow-up questions for each of A1-A3 and asks them to re-evaluate. The maximum number of such turnarounds is predefined by the user. Additionally, three independent deep research models with web-search tools are used to directly search for eligible trials online. All results are integrated by the result agent, and non-consensus trials are flagged for human revision. The agents mentioned above are Large Language Models prompted to address specific tasks. For the reasoning models A1-A3 and A4, we use either Gemini 3 Pro or DeepSeek Reasoning with temperature = 0.7, top p = 0.9; for the result agent we use Gemini 3 Pro with temperature = 0.5, top p = 0.6.
