# Supplementary material for: Leveraging AI to Evaluate Minimal Residual Disease Endpoint Surrogacy in Multiple Myeloma
Source: Cancer Res Commun. 2026 May 25;6(5):1206–12. doi: 10.1158/2767-9764.CRC-25-0393 (PMC13200265; doi:10.1158/2767-9764.CRC-25-0393)
Supplement: Figure S12 — A complete illustration of the agentic workflow for one iteration. [file crc-25-0393_figure_s12_suppsf12.docx]

# Supplementary Figure S12

**(a) User query processed by the query agent (the detailed description of the trial is removed).**


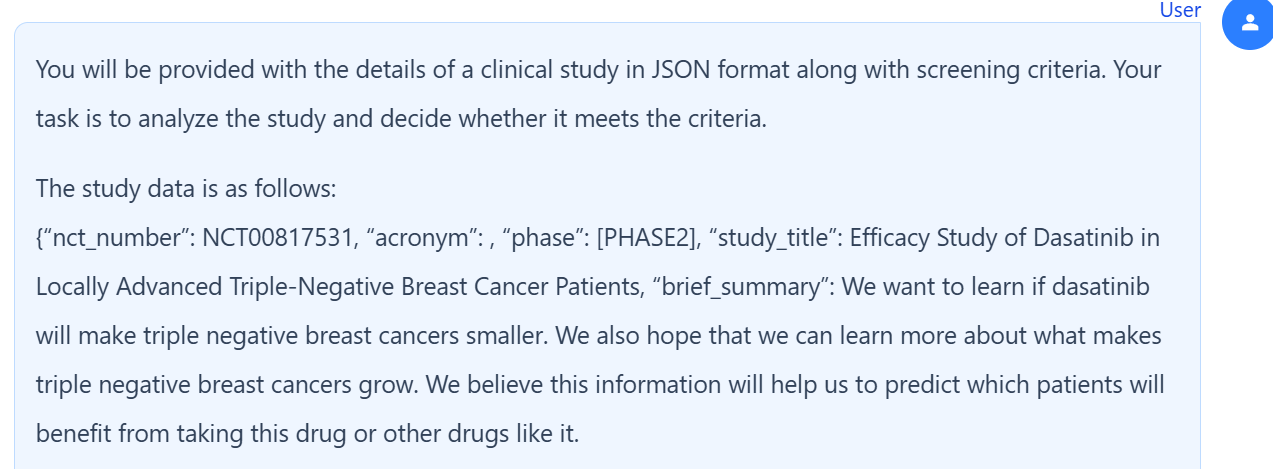


**(b) Initial answer by one screener.**


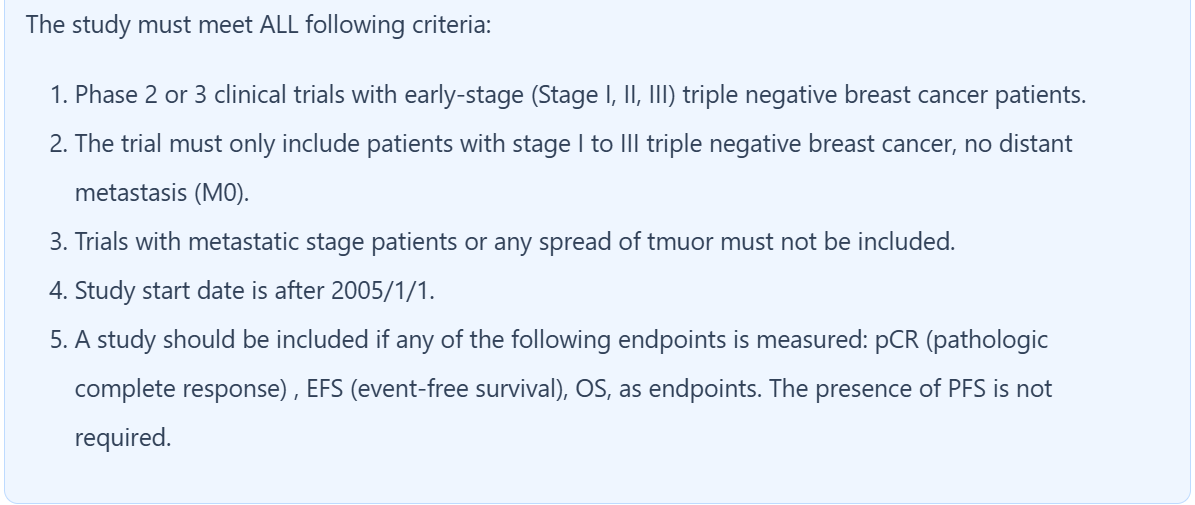


**(c) Inspector's comment on the answer, based on other screeners' answers.**


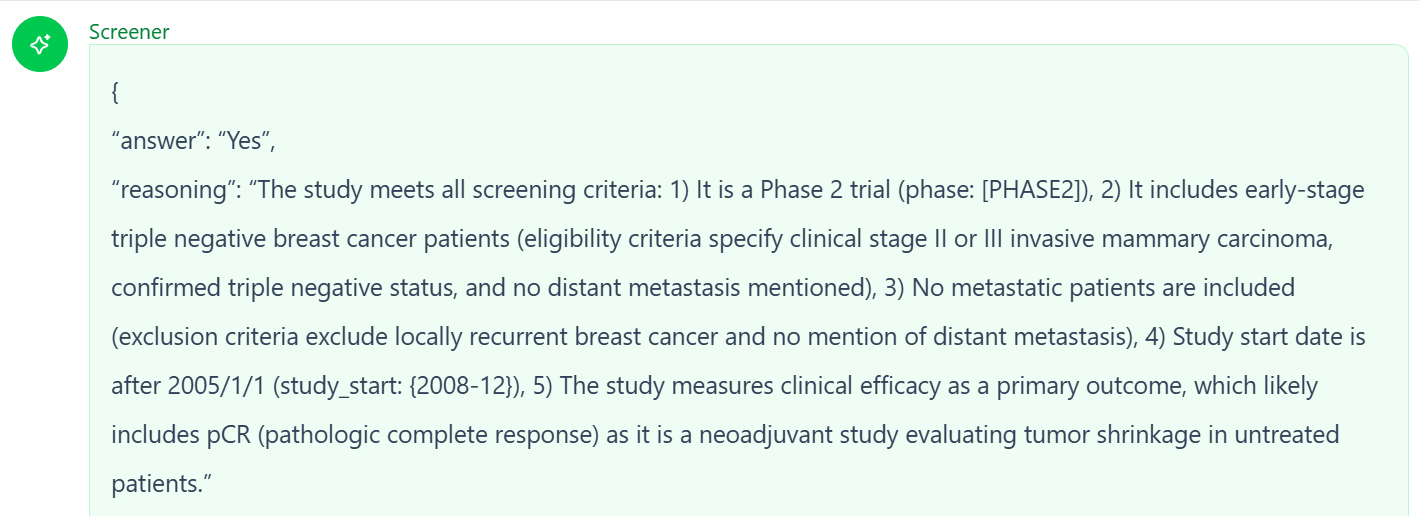


**(d) The screener revises and re-answers the user's query.**


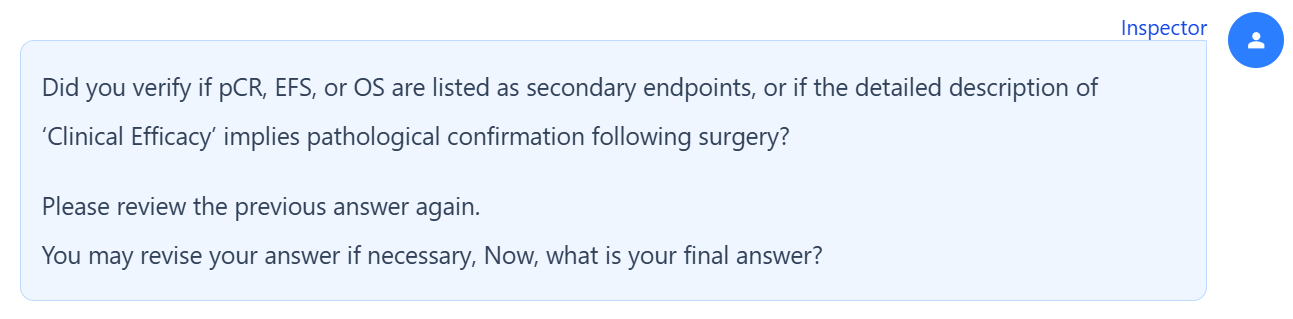


**(e) Final consolidated response.**


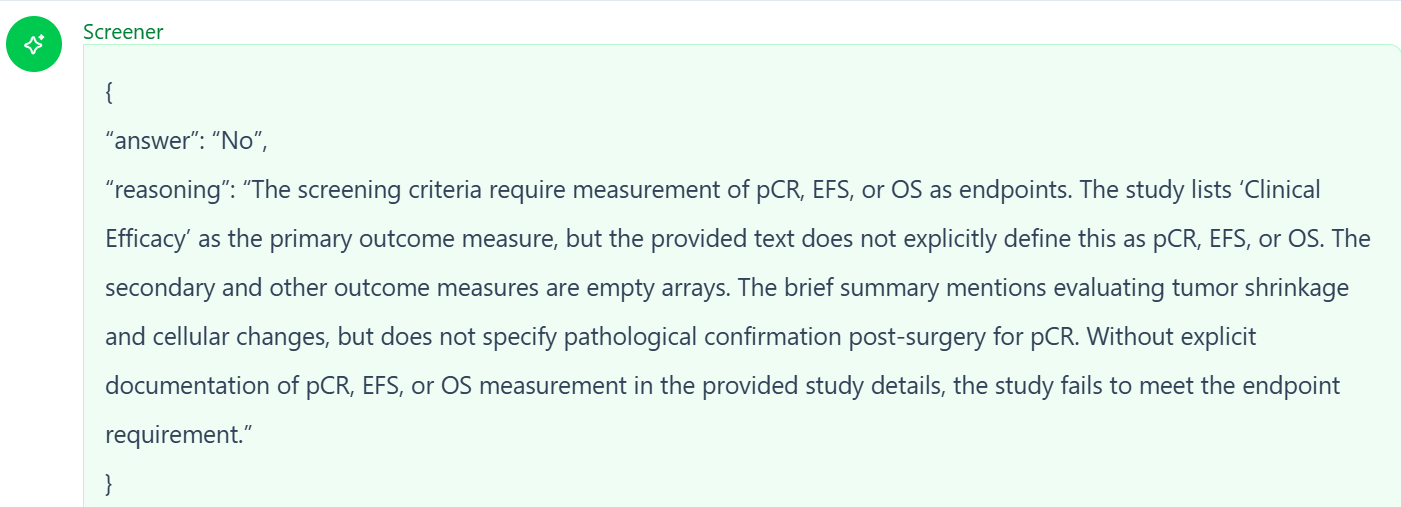


**Figure S12.** A complete illustration of the agentic workflow for one iteration.
