# Supplementary material for: Leveraging AI to Evaluate Minimal Residual Disease Endpoint Surrogacy in Multiple Myeloma
Source: Cancer Res Commun. 2026 May 25;6(5):1206–12. doi: 10.1158/2767-9764.CRC-25-0393 (PMC13200265; doi:10.1158/2767-9764.CRC-25-0393)
Supplement: Descriptive notes for supplement [file crc-25-0393_descriptive_notes_for_supplement_suppsm.docx]

**Supplementary materials for “Leveraging AI to Evaluate Minimal Residual Disease Endpoint Surrogacy in Multiple Myeloma”**

**Additional results and sensitivity analysis for the first task**

For trial-level association between PFS log(HR) versus MRD- CR log(OR), a sensitivity analysis using only 10^{-5} MRD measurement sensitivity was performed, using both sample sizes and PFS log(HR) variances as weights, see *Supplementary* *Figure S1,S2*.

*Supplementary Figure S3-S5* presented the association analysis of OS log(HR) and ORR log(OR) against MRD- CR log(OR). For OS analysis, only a total of 14 studies were included because the OS data were not mature for OCTANS, TOURMALINE-MM2, PLEIADES, TOURMALINE-MM3 [20, 17, 6, 15] at the date of analysis. Similarly, GEM2012MENOS65 [13] was excluded when analyzing ORR because of missing information on ORR.

**Sensitivity analysis of trial-level associations by MRD measurement method and MRD measurement timings**

We also conducted a sensitivity analysis based on the method and timing of MRD assessment, see *Supplementary Figure S8, S9*. We observed that many studies evaluated MRD negativity at multiple time points, resulting in heterogeneous assessment schedules across trials. To ensure consistency, we restricted this analysis to the eight studies that explicitly reported MRD assessment at the time when patients were suspected to have achieved complete response. Of these eight trials, one study (PERSEUS) reported progression-free survival (PFS) results only; therefore, seven trials were included in the overall survival (OS) analysis. The results from this subgroup were consistent with those of the primary analysis. For example, the PFS association analysis yielded an $R^{2}=0.77$, which is comparable to the primary analysis result ($R^{2}=0.71$).

**AI agentic workflow**

We provide the workflow in *Supplementary Figure S11* and provide an example of the chat history of our workflow in one specific run on triple-negative breast cancer in *Supplementary Figure S12.*
